# Editorial: *Porphyromonas gingivalis*: molecular mechanisms of invasion, immune evasion, and dysbiosis

**DOI:** 10.3389/fcimb.2023.1289103

**Published:** 2023-09-29

**Authors:** Aditi Chopra, Yuka Shiheido-Watanabe, Joerg Eberhard

**Affiliations:** ^1^ Department of Periodontology, Manipal College of Dental Sciences, Manipal, Manipal Academy of Higher Education, Manipal, Karnataka, India; ^2^ Department of Cardiovascular Medicine, Tokyo Medical and Dental University (TMDU), Tokyo, Japan; ^3^ Sydney Dental School and the Charles Perkins Centre, Faculty of Medicine and Health, The University of Sydney, Sydney, NSW, Australia

**Keywords:** *Porphyromonas ginigivalis*, oral microbiome, oral bacteria, oral microbiology and immunology, dysbiosis, inflammation and immune dysfunction, microbiology, oral-systemic association

*Porphyromonas gingivalis* (*P. gingivalis*), formerly known as *Bacteroides gingivalis*, is a Gram-negative, asaccharolytic, obligate, capnophilic, anaerobic, rod-shaped oral bacteria, primarily associated with the pathogenesis and progression of periodontal disease (How et al., 2016). *P. gingivalis* has been linked to many chronic systemic diseases in humans such as inflammatory bowel disease, ulcerative colitis, cardiovascular diseases, Alzheimer’s disease, diabetes, rheumatoid arthritis, preterm birth low birth weight, and cancers (Peng et al., 2022).

Many unique molecular mechanisms make this ‘keystone pathogen’ a potential microbe for numerous systemic diseases. One of the key factors is its ability to evade the host’s defense mechanisms and induce immune subversion. It escapes phagocytosis by modulating the immune cells like neutrophils and macrophages. It utilizes these cells for its nutrition, translocation, and survival in the body. It can even survive in extreme anaerobic environments at distant organ sites and induce inflammation (How et al., 2016). *P. gingivalis* is recognized as a ‘master of polymicrobial synergy and dysbiosis’, as it engineers its environment and modifies its nutritional demand to survive and persist in the host. The ‘inflammophilic’ and acid-resistant nature of *P. gingivalis* helps it to survive in the gut and blood circulation. It is noted that approximately 10^8^–10^10^ *P. gingivalis* are swallowed each day. Since this microbe can tolerate the acidity of the stomach, it can reside and proliferate in the gastrointestinal tract and spread to other organs. *P. gingivalis* entry into the host helps in the maturation and growth of other microorganisms within the body. It can alter the growth of other microbes and induce dysbiosis in distant organ systems by indirectly sending the short-range paracrine signaling molecules into its environment and enhancing quorum sensing (Chopra et al., 2020). The translocation of oral microbes into the gut provides a potential role of the oral cavity in gut dysbiosis (Olsen and Yamazaki, 2019). Many powerful virulence factors of *P. gingivalis* such as gingipains (cysteine proteases), capsule, lipopolysaccharide (LPS), fimbriae, nucleoside diphosphate kinase (NDK), ceramide, and outer membrane proteins (OMPs) are identified as modulators of systemic inflammation, dysbiosis, tumorigenesis, and immune response evasion. These virulence factors (also known as adhesins) modulate the various signaling pathways, host receptors, and cells of inflammation such as the complement system, Toll-like receptors (TLRs), neutrophils, macrophages, dendritic cells, B-cells and T-cells (Th1/Th2/Th17) (Zheng et al., 2021).

Our Research Topic highlighted some novel pathogenic mechanisms that confirm the role of *P. gingivalis* in tumorigenesis and cardiovascular disease. A review article in our Research Topic by Chow et al. discussed the role of gingipain R in the process of citrullination. Citrullination relies on the interplay between two virulence factors of this bacterium, namely gingipain R and the bacterial peptidyl arginine deiminase. Gingipain R cleaves the host proteins and exposes the C-terminal arginine for peptidyl arginine deiminase to generate citrullinated proteins. The citrullinated proteins get deposited in the host tissues and induce organ dysfunction. These citrullinated proteins have been identified in the periodontal tissues, synovial tissues and joints, atherosclerotic plaques, and neurons of patients with Alzheimer’s disease, rheumatoid arthritis, and cardiovascular disease. The review also highlighted how inhibiting the gingipain and peptidyl arginine deiminase would help to manage periodontitis and other systemic diseases involving bacterial citrullination.

Another review by Ruan et al. also found that the gingipains and OMPs of *P. gingivalis* affect the functioning of the cardiovascular system by modulating the host immune response and increasing the process of atherosclerosis. The authors appraised the existing clinical and animal studies and summarised the role of *P. gingivalis* in the progression of atherosclerosis. The review discusses the mechanisms by which *P. gingivalis* escapes the host immune system and circulates in the blood and lymphatic circulation. The review also discusses how *P. gingivalis* can colonize the arterial vessel walls and injure the endothelium thereby directing a local inflammatory response and fibrogenesis. It can even impair the physiologic remodeling of the cells of the blood vessels and is known as a potential risk factor for cardiovascular disease. *P. gingivalis* has been identified in various blood vessel in the body, including the blood vessels of the heart, myocardium, cardiac muscles, and cardiac valves. Its ability to induce acute phase response and autoimmune antibodies link it to altered lipid profile, thrombogenesis, and atherosclerosis. The authors also provide new insights for preventing and treating atherosclerosis by suppressing periodontal pathogenic bacteria.

Another study by Phillips et al. noted the role of the OMPs in the physiologic remodeling of the uterine spiral arteries and how it can lead to adverse pregnancy outcomes. The study found that OMPs and gingipains alter the VSMC phenotype and this change dysregulates the VSMC plasticity of the uterine spinal artery. *P. gingivalis* can also induce physiologic remodeling of the uterine spinal artery by inducing apoptosis and increasing the release of plasminogen activator inhibitor-1, platelet activation factors, and various trypsin enzymes from the inflammatory cells. The OMPs components have been identified to have a high affinity toward the aortic smooth muscle cells, whereby they increase the colonization of *P. gingivalis* within the blood vessels and alter its function.

Previous studies have identified *P. gingivalis* as a potential risk factor for cancers of the oral cavity, pancreas, prostate, rectum, colon, and stomach (Peng et al., 2022). The link between tumorigenesis and *P. gingivalis* is attributed to the ability of this unique microbe to provide an inflammatory milieu, modulate the cell cycle, and accelerate tumor cell evasion. Our Research Topic also has two studies that highlight the role of *P. gingivalis* in prostrate and oesophageal cancer. An article by Groeger et al. investigated the expression and signaling pathway of programmed death ligand 1 (PD-L1) in a prostate cancer cell line after infection with *P. gingivalis*. The authors noted that the PD-L1 signaling pathway was upregulated in prostate cancer cells after infection with both viable and heat-killed *P. gingivalis* membrane fractions. The peptidoglycan of *P. gingivalis* also up-regulated PD-L1 signaling and this was mediated by the NOD1/NOD2 signaling pathway. No upregulation was detected after treatment of the prostrate cells with *P. gingivalis* LPS. The role of *P. gingivalis* and cancer is also confirmed by another study in our Research Topic by Jia et al. where the authors found that *P. gingivalis* can upregulate the proliferation and migration of normal human oesophageal epithelial cells and lead to the development of aneuploid cells. The malignant transformation of healthy oesophageal epithelium to cancerous cells by *P. gingivalis* occurs via the ‘sonic hedgehog pathway’.

These intriguing pieces of evidence confirm the role of *P. gingivalis* in the onset and progression of many systemic diseases. This Research Topic strengthens the link between the oral microbiome and systemic health. The connection between our oral cavity to the rest of the body is vital and the intricate pathogenic mechanism explaining the oral-systemic link should be explored and researched! With the advancement in bioinformatics and metagenomic technology, an in-depth analysis of how oral bacteria like *P. gingivalis* affect the functioning of various organ systems is important. Since there is a rise in the availability of genomic information and metagenome-assembled genomes (MAGs) for different oral microbes, the possibilities for the whole genomic analysis of several aspects of evolution and understanding of the unique ability of *P. gingivalis* is now a possibility. Thus, comparative genomics studies should explore how *P. gingivalis* acquired its features to become a ‘keystone pathogen’. This would help to understand the role of *P. gingivalis* in modulating various oral and systemic diseases at the genomic and transcriptomic levels. The relationship between the genetic diversity, pathogenesis, evolution, and interaction of *P. gingivalis* with other microbes, including viruses and fungi, is another vital area for future research. The interaction of *P. gingivalis* with other putative pathogens of the human body can be explored to understand its role in cross-contamination, disease transmission, and progression of inflammation in the host.

## Author contributions

AC: Conceptualization, Formal Analysis, Resources, Supervision, Validation, Visualization, Writing – original draft, Writing – review & editing. YS-W: Conceptualization, Supervision, Validation, Writing – original draft, Writing – review & editing. JE: Conceptualization, Supervision, Visualization, Writing – original draft, Writing – review & editing.
